# Tumor Stroma Ratio and Its Significance in Locally Advanced Colorectal Cancer

**DOI:** 10.3390/curroncol29050263

**Published:** 2022-05-03

**Authors:** Luz Sullivan, Richard R. Pacheco, Michel Kmeid, Anne Chen, Hwajeong Lee

**Affiliations:** Department of Pathology, Albany Medical Center, Albany, NY 12208, USA; sullivl1@amc.edu (L.S.); pachecr1@amc.edu (R.R.P.); kmeidm@amc.edu (M.K.); chena6@amc.edu (A.C.)

**Keywords:** colorectal cancer, tumor stroma ratio, tumor microenvironment, tumor budding, immune cells

## Abstract

Colorectal cancer is the third leading cause of cancer-related death, and its incidence is rising in the younger patient population. In the past decade, research has unveiled several processes (underlying tumorigenesis, many of which involve interactions between tumor cells and the surrounding tissue or tumor microenvironment (TME). Interactions between components of the TME are mediated at a sub-microscopic level. However, the endpoint of those interactions results in morphologic changes which can be readily assessed at microscopic examination of biopsy and resection specimens. Among these morphologic changes, alteration to the tumor stroma is a new, important determinant of colorectal cancer progression. Different methodologies to estimate the proportion of tumor stroma relative to tumor cells, or tumor stroma ratio (TSR), have been developed. Subsequent validation has supported the prognostic value, reproducibility and feasibility of TSR in various subgroups of colorectal cancer. In this manuscript, we review the literature surrounding TME in colorectal cancer, with a focus on tumor stroma ratio.

## 1. Introduction

Colorectal cancer (CRC) is the second most common cause of cancer and the third leading cause of cancer-related death [1]. In the past decade, the incidence of CRC in individuals younger than 50 years has been increasing, leading to a growing CRC-related healthcare burden [1]. Management and treatment of these malignancies is largely determined by histopathologic diagnosis. Similarly, histologic morphology can aid in prognostication by predicting tumor behavior and patient outcomes. The microscopic field can be simplistically divided into two segments: the neoplastic cells and the remaining stroma. Historically, attention has been mainly directed towards understanding the neoplastic cells with little regard for the stroma within which they reside. However, as the understanding of these complex diseases matures, efforts have been redirected towards evaluating the entire tumoral milieu or tumor microenvironment (TME). These constituents include inflammatory and immune cells, relative hypoxia and resultant activation of hypoxia-inducible factor-1 (HIF-1), stromal make up and extracellular components including extracellular matrix (ECM), soluble factors and proangiogenic molecules, particularly vascular endothelial growth factor (VEGF) [2,3,4,5,6,7,8,9,10]. A recent area of focus is the amount of stroma relative to the neoplastic cells or tumor stromal ratio (TSR) [11]. In this study, we review the relevant literature of the TME in CRC and highlight recent findings surrounding TSR.

## 2. Current Staging Model and Drawbacks

The current TNM staging system stratifies patients based on the extent of cancer spread. Treatment regimens vary greatly among these stages, with local tumor excision at one end of the spectrum and neoadjuvant therapy with (procto)colectomy and adjuvant therapy at the other end. Although the TMN system provides a standardized guidance for management, outcomes vary depending on several factors [12,13]. For example, the expected benefit of adjuvant chemotherapy is higher in average-risk stage III patients compared to average-risk stage II patients. However, the decision for adjuvant chemotherapy gets more complex when stage II tumors show high-risk features such as T4, poorly differentiated/undifferentiated histology (excluding microsatellite unstable (MSI-H) tumors), lymphovascular invasion, perineural invasion, tumor budding, bowel obstruction, perforation, close, indeterminate or positive margins or inadequately sampled lymph nodes (less than 12 lymph nodes). Current National Comprehensive Cancer Network (NCCN) recommendations allow for either 3 or 6 months of adjuvant chemotherapy or simple observation in stage II patients with these high-risk features, whereas chemotherapy is recommended for all patients with stage III disease and regimens depend on the additional risk stratification [14].

Also, microsatellite instability (MSI) is an important factor to consider when determining the benefit from adjuvant therapy. For example, MSI-H CRCs tend to have favorable prognosis irrespective of tumor differentiation, whereas fluorouracil-based adjuvant chemotherapy may not give survival advantage to patients with stage II MSI-H tumors [15]. Inconsistencies such as these highlight the limitations of extent-based staging and the need for a robust biomarker.

## 3. Biomarkers

The recent literature has investigated the utility of biomarkers and their role in patient stratification [16]. Early studies have found that certain patient demographics, tumor characteristics and aspects of surgery were linked to worse outcomes [12]. The severity of the aforementioned high-risk features and their prognostic value is reflected in the published guidelines of various professional bodies including the Society of Clinical Oncology and the European Society of Medical Oncology [17,18]. In addition, for locally advanced diseases, MSI status and circulating tumor DNA (ctDNA) have proven useful in predicting response to chemotherapy as well as association with disease-free survival (DFS) [15,19]. *RAS*, *BRAF*, HER2/neu and MSI status are all recommended for patients with metastatic CRC [14,20,21]. Also, several multigene and immune assays have been developed in an attempt to fine tune the current risk stratification and promote precision medicine [19].

## 4. Tumor Microenvironment in Colorectal Cancer

Histologic biomarkers focus on morphologic aspects of the tumor and its composition rather than its anatomical location and behavior. The substance of the tumor is comprised not only of neoplastic cells but also surrounding stroma which includes immune cells, fibroblasts, signaling molecules and ECM. These components collectively make up the TME. Recent literature about the TME has shed light on CRC tumorigenesis and the complex interactions between tumor cells and the surrounding stroma [2,3,4].

On routine histologic assessment, pathologists can recognize prognostically valuable aspects of the TME, such as variations in tumor stroma, the presence of tumor budding and host inflammatory response [5]. Survival analyses have demonstrated that these histologic parameters may outperform conventional TNM staging [6,7]. Among these new features, the proportion of tumor stroma relative to tumor cells has been identified as an important determinant of tumor progression, especially in CRC [11].

### 4.1. Stroma

Stromal cells drive tumor progression via the secretion of soluble factors, modulation of the ECM and stimulation of cell migration [22]. Stromal cells provide a scaffold for tumoral cells to grow, supply survival signals including insulin growth factor and CXCL12 and lay down extracellular elements such as collagen, proteoglycans, glycoproteins and integrins [23]. This ECM deposition creates a protective environment for tumor cells by increasing stromal density and tension, which may prevent the efficacy of anticancer agents such as biologics and chemotherapy [22,23].

### 4.2. Epithelial Mesenchymal Transition

Through secretion of chemokines and growth factors, the TME enables neoplastic epithelial cells to undergo a process referred to as epithelial mesenchymal transition. Tumor cells then acquire a mesenchymal phenotype leading to invasive potential enhanced migration and subsequent disease progression [24,25]. Similarly, the malignant cells transform the surrounding environment by changing the composition of the stroma [22].

### 4.3. Immune Cells

As part of the interaction between the tumor cells and the tumor bed, immune cells are thought to represent the antitumoral host response [26]. T lymphocytes are one of the major type of cells present in tumors [24]. CD8 T lymphocytes exert cytotoxic actions and CD4 T lymphocytes activate natural killer cells as well as antigen presenting cells. Together, these actions of CD8 and CD4 T cells control tumor growth. Macrophages, as part of innate immunity, are mobilized in response to stimuli from TME and activate inflammatory responses through different mechanisms. The prognostic value of immune cells within and adjacent to the tumor has been validated by multiple survival studies and different cell populations have been characterized [27].

### 4.4. Tumor Budding

Another well-studied component of TME is small groups of tumor cells at the invasive front, defined as tumor budding (TB). TB has been linked to adverse oncologic outcomes in CRC such as decreased survival and an increased risk for lymph node metastasis [28]. As a high-risk feature, TB was recently incorporated into guidelines for locally advanced CRC by the European Society for Medical Oncology (ESMO) [18].

### 4.5. Carcinoma Percentage

In 2007, Mesker et al. were the first to publish on the association between carcinoma percentage (CP) relative to stroma and CRC progression [29]. The authors compared patients with high CP tumors to those with low CP tumors and reported lower overall survival (OS) and DFS in the low CP group [29]. These findings suggest stroma plays an active role in CRC progression and resulted in the development of a scoring system to calculate the amount of stroma as a ratio [30].

## 5. Tumor Stroma Ratio

Tumor stroma ratio (TSR) is defined as the percentage of the neoplastic cell component relative to the stroma in tumor tissue [31]. TSR is determined by evaluation of hematoxylin and eosin (H&E) stained tissue sections and is considered a biomarker derived from the TME [32].

### 5.1. Scoring Protocol

**Figure 1:**
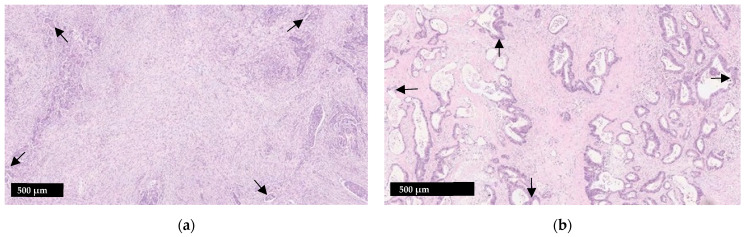
Illustration of the tumor stroma ratio (TSR) (**a**) Stroma-high tumor; (**b**) Stroma-low tumor. When assessing adequacy of a visual field, tumor cells should be present at four sides which are roughly 90 degrees from one another (arrows). Smooth muscle, lymphoid follicles and large vessels with thick muscular walls should be disregarded.

**Figure 2:**
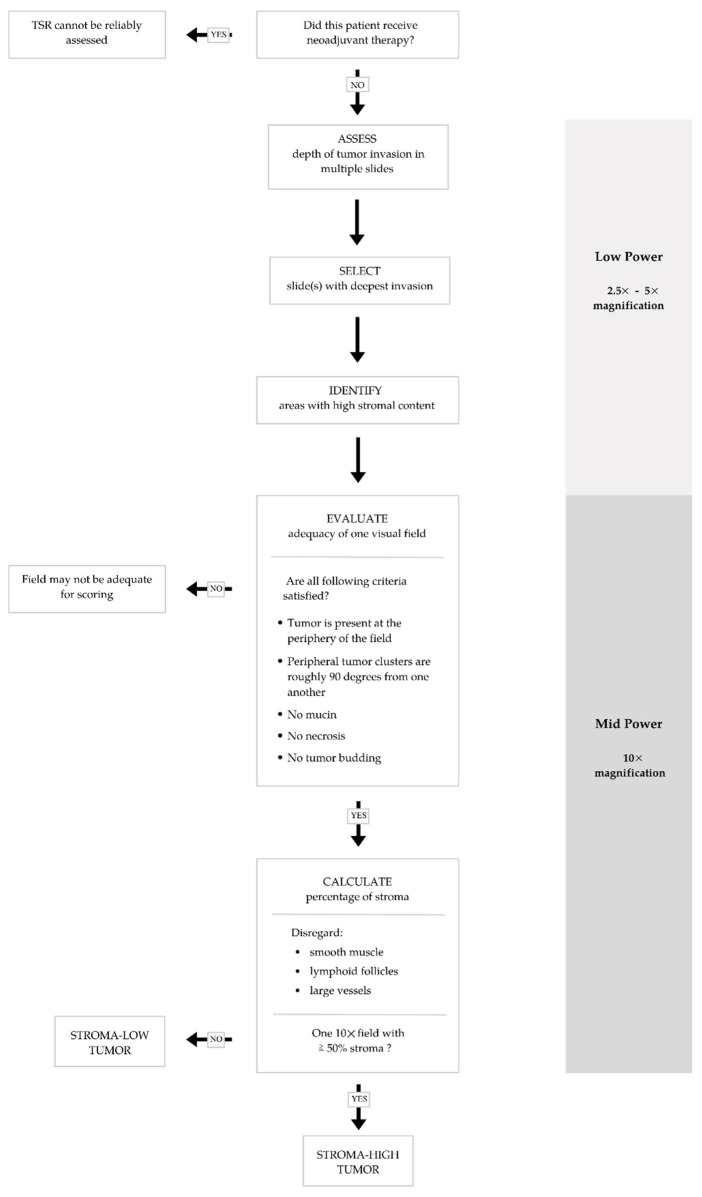
Flow chart summarizing steps to ensure accurate and reproducible evaluation of tumor stromal ratio (TSR) in adequate visual fields.

Various methodologies to estimate the TSR have been proposed [16,29,30]. The protocol developed by van Pelt et al. has high prognostic impact and can be easily implemented in daily practice [16]. TSR is assessed on the same slides used to determine the T stage. Therefore, the slide(s) with the deepest invasion is selected for evaluation. Next, the ×2.5 or ×5 objective is used to identify areas with the highest percentage of stroma. These areas are evaluated for adequate microscopic fields, which was determined to be one ×10 field (approximately 2.54–2.80 mm^2^) containing both tumor cells and stroma. Additionally, tumor clusters need to be located at four sides of the microscopic field and approximately 90 degrees from one another. For example, if tumor cells are identified at the 12:00 position, at minimum there must also be tumor cells at the 3:00, 6:00 and 9:00 positions (Figure 1), respectively. Only adequate fields are used to calculate stromal percentage, which is reported in 10% increments. If one 10× field with greater than 50% stroma is identified, the tumor is deemed stroma-high. If no such field is identified, the tumor is deemed stroma-low (Figure 2) [16]. Previous studies have shown that a cutoff of 50% allows for the maximum discriminative power [29].

Other microscopically evident structures such as smooth muscle tissue, lymphoid follicles and large vessels are considered part of the native constituents of the large bowel and should be left out of the microscopic field or disregarded when scoring. Similarly, mucin, necrosis and tumor budding can interfere with scoring and should be avoided as well [30]. TSR scoring is not applicable to specimens from CRC patients who received neoadjuvant therapy as stromal composition may change following neoadjuvant therapy [30].

### 5.2. Interobserver Variability and Intratumor Heterogeneity

Since TSR is a histologic parameter that can be easily assessed by routine microscopy alone, its reproducibility and feasibility have been scrutinized. Souza et al. reported high interobserver agreement among pathologists scoring TSR in CRC [33]. These results have been validated with several studies reporting moderate to high interobserver agreement in TSR assessment (Cohen’s kappa, range 0.42–0.85) [32,34,35,36].

A similar concern focuses on the effect of intratumoral heterogeneity. Eriksen et al. studied this in a cohort of 43 stage II CRC specimens. Using both conventional microscopy and a stereology platform, the authors concluded that TSR can be semi-quantitatively assessed in a consistent manner when a slide with deepest invasive tumor and low intratumoral heterogeneity is selected [35].

### 5.3. Prognostic Value

Tumor associated stroma plays an active role in tumor invasion and metastasis. In a meta-analysis including 4238 patients with solid tumors, the relationship between TSR and prognosis was explored. The authors found that patients with low TSR (stroma-high) were at increased risk of shorter OS and DFS, advanced clinical stage, increased depth of invasion and lymph node metastasis [37]. These findings have been reproduced in subsequent studies which found that stroma-rich tumors had worse outcomes [38,39].

However, the association between TSR and outcomes is less clear in CRC patients. Several studies observed little or no support for TSR as a prognosticator [32,34,40]. For example, the Dutch T1 CRC Working Group studied the prognostic value of TSR in 261 T1 CRC tumors that were surgically removed with lymph node dissection. After a mean follow up of 43 months, low TSR (stroma-high) was not associated with nodal disease or recurrence [34]. Similarly, Smit et al. evaluated TSR in a cohort of 246 patients with stage II or III CRC who underwent surgical intervention. After a median follow-up of 47 months, TSR was found to be an independent prognosticator for DFS but not for OS [32]. The same findings were reported by Zhang et al. who studied the prognostic value of TSR across all CRC stages. Their cohort included 84 patients with stage I or II disease and 63 patients with stage III or IV. After a median follow-up of 49 months, TSR was not predictive of recurrence free survival (RFS) or OS [40].

Of note, the studies reporting no association between TSR and outcomes all had relatively short follow up, ranging from 43 months to 49 months. However, studies with longer follow up intervals found that TSR was a useful prognosticator of survival and other outcomes [11,29,41,42,43,44]. For example, after 7 years of follow up, Eriksen et al. found that TSR was an independent prognostic marker of RFS and OS along with age (cutoff 73 years), T stage and tumor perforation in a cohort of 573 patients [42]. Similarly, after a minimum of 5 years follow up, van Wyk et al. documented that TSR was predictive of cancer-specific survival in a cohort of 952 patients with operable CRCs (stage I: 131, II: 445, III: 355, IV: 21 patients) [43].

Although the results are far from definitive, these findings suggest TSR is most useful in long-term predictions. Nevertheless, the utility of TSR as a prognostic marker in CRC has been presented and discussed by several professional societies to include the TNM Evaluation Committee, the Union for International Cancer Control (UICC) and the College of American Pathologists (CAP). As initial studies were retrospective, further validation by prospective studies was recommended [45].

In 2018, a multicenter prospective cohort study was initiated in the Netherlands with two objectives. The first is to evaluate interobserver variability through web-based training. The second was to validate the prognostic power of TSR in a large, 1500-patient population. This study is currently in progress and the results are expected to be presented in 2023 [45].

### 5.4. Tumor Stroma Ratio and Tumor Characteristics

A few studies evaluated associations between TSR and histopathologic tumor characteristics in CRC. Stroma-high CRC tended to have higher T and N stage, resection margin positivity, peritoneal involvement and infiltrative growth at the invasive front and TB, whereas tumor necrosis was more common in stroma-low CRCs (high TSR) [38,42,44]. MSI-H CRCs tended to show high TSR (stroma-low) [44]. There was no difference in gender of the patients, tumor location (colon vs. rectum), tumor differentiation, venous invasion, tumor perforation and local or systemic inflammatory response between the stroma-high and stroma-low groups [38]. Conflicting data exist regarding the associations between age vs. stroma amount [38,46].

### 5.5. Resistance to Therapy

As is the case with other intrinsic tumor features such as hypoxia, pH and vascular shunting, TSR may contribute to chemoresistance [47]. Hagenaars at al observed that, when compared to their stroma-high counterparts, patients with stroma-low breast tumors were 2.46 times more likely to have a complete response after neoadjuvant therapy [48]. Similar observations have been reported in esophageal carcinoma, suggesting the deleterious effect of stroma in gastrointestinal tumors [49].

In a recent study comparing rectal cancer biopsies, Liang et al. observed a similar trend as patients with high stromal content biopsies were less likely to respond to neoadjuvant treatment. Likewise, the amount of stroma in the pre-treatment biopsy was inversely correlated with the degree of tumor regression [50].

## 6. Other Biomarkers

### 6.1. Extracellular Elements

The composition of tumoral stroma also has prognostic value. An earlier study by Goncalves-Ribeiro et al. compared the pre-treatment and post-treatment genetic profiles of tumoral stroma and glands of in rectal cancer specimens. They identified combined fibronectin and collagen 3A1 expression as an independent predictor of resistance to treatment [51]. These findings highlight the value of stromal analysis and suggest that not only quantity but also the composition of stroma may influence responsiveness to treatment.

ECM is a complex formed by proteins, glycoproteins and proteoglycans. In addition to providing structural support, the ECM is integral to cell proliferation, migration and growth. A key component of the ECM are matrix metalloproteases (MMP), which mediate ECM degradation. MMPs are crucial in the molecular communication between stroma and tumor cells, regulation of VEGF bioavailability and vascular permeability which can promote tumoral progression and invasiveness [24,52].

### 6.2. Inflammatory Cells

Immune cells comprise a large component of the TME and similarly provide prognostic value. Ravensbergen et al. assessed both TSR and immune cell infiltration as predictors of responsiveness to immune checkpoint inhibitor (ICI) therapy using the colon cancer database in the Cancer Genome Atlas (TCGA) [36]. Response to ICI therapy was greater in tumors with high immune cell infiltration regardless of the amount of stroma. However, when the immune evasion model (Tumor Immune Dysfunction and Exclusion algorithm) was employed, the authors noticed that tumors with high stromal content had decreased responsiveness to ICI therapy. This finding suggests that the stroma may be directly involved in immune evasion, and combined TSR and immune cell infiltration may be a superior predictor of responsiveness to ICI therapy [36].

Evaluation of inflammatory cells can also provide information on tumor progression and patient prognosis. Studies have shown that the type and density of immune cells are determinants of tumor progression [53]. The inflammatory reaction at the invasive front can be scored although the scoring system has only been validated for research purposes [54]. Hynes et al. analyzed peritumoral inflammation and TSR in patients with stage II/III colon cancer. The predictive power was enhanced when these two biomarkers were combined as a single fibroinflammatory score. Patients with high stroma and low inflammatory infiltrate displayed the worst prognosis and the authors recommended incorporating both markers for prognostication [5].

### 6.3. Tumor Budding

In addition to marked inflammatory cell infiltrate, TB has been shown to have prognostic value as it is associated with recurrence, metastasis, DFS and OS in CRC [26,44,55,56,57]. Interestingly, this powerful prognosticator may be related to TSR. Eriksen et al. analyzed the prognostic value of TSR and TB in stage II colon cancer and reported an association between high TB and increased tumor stroma. Given their association, the authors compared the two and found that TSR outperformed TB as a marker for RFS and OS [42]. These results were supported by the findings of Smit et al. who observed that both TSR (stroma-high) and TB were prognostic of DFS, however TSR was more reproducible and considered a superior biomarker (kappa = 0.83) compared to TB (kappa = 0.47) [32]. Nonetheless, conflicting observations exist in the literature [40,43,58].

Additionally, age is related to TSR and TB. In a study exploring the predictive value of TB and TSR for recurrence and death in elderly patients with stage I colon cancer, authors found that the number of tumor buds and the amount of stroma increased at older ages (>68 years). Similarly, TSR and TB were stronger prognostic factors in the elderly population. These findings demonstrate the possible effects of age on TSR and the TME [46].

## 7. Future Applications

Analysis of the TME and TSR has numerous valuable prognostic applications; however, their assessment is limited to resected CRC specimen. Ravensbergen et al. constructed a novel “stroma–epithelial gene signature ratio” that can be applied to liquid biopsy using a proteomics panel containing key proteins involved in TME of CRC. Using the TCGA COAD cohort (*n* = 333) as a discovery set, the authors demonstrated that high stroma tumors had enriched stromal gene signatures. The signature ratio was predictive of OS in both the discovery and validation (*n* = 229) cohorts [59].

## 8. Conclusions

The significance of TME in tumor progression, prognosis and responsiveness to therapy is increasingly recognized. TSR is a relatively new and promising histologic biomarker with potential as a robust prognosticator in CRC. Further collaborative efforts are warranted to verify its utility as a prognostic marker in daily practice. Whether this histologic biomarker would outweigh or become an adjunct to ctDNA monitoring for minimal residual disease awaits further investigation.
